# Enhanced Wound Healing Potential of Primary Human Oral Fibroblasts and Periodontal Ligament Cells Cultured on Four Different Porcine-Derived Collagen Matrices

**DOI:** 10.3390/ma13173819

**Published:** 2020-08-29

**Authors:** Zhikai Lin, Cristina Nica, Anton Sculean, Maria B. Asparuhova

**Affiliations:** 1 Laboratory of Oral Cell Biology, Dental Research Center, School of Dental Medicine, University of Bern, Freiburgstrasse 3, 3010 Bern, Switzerland; zhikai.lin@zmk.unibe.ch (Z.L.); cristina-gabriela.nica@students.unibe.ch (C.N.); 2 Department of Periodontology, School of Dental Medicine, University of Bern, Freiburgstrasse 7, 3010 Bern, Switzerland; anton.sculean@zmk.unibe.ch; 3 Department of Periodontology, Shanghai Ninth People’s Hospital, School of Medicine, Shanghai Jiaotong University, Zhizaoju Road 639, Shanghai 200011, China

**Keywords:** soft tissue regeneration, biomaterials, xenografts, connective tissue grafts, growth factors, wound scratch, gene expression

## Abstract

Xenogenic collagen-based matrices represent an alternative to subepithelial palatal connective tissue autografts in periodontal and peri-implant soft tissue reconstructions. In the present study, we aimed to investigate the migratory, adhesive, proliferative, and wound-healing potential of primary human oral fibroblasts (hOF) and periodontal ligament cells (hPDL) in response to four commercially available collagen matrices. Non-crosslinked collagen matrix (NCM), crosslinked collagen matrix (CCM), dried acellular dermal matrix (DADM), and hydrated acellular dermal matrix (HADM) were all able to significantly enhance the ability of hPDL and hOF cells to directionally migrate toward the matrices as well as to efficiently repopulate an artificially generated wound gap covered by the matrices. Compared to NCM and DADM, CCM and HADM triggered stronger migratory response. Cells grown on CCM and HADM demonstrated significantly higher proliferative rates compared to cells grown on cell culture plastic, NCM, or DADM. The pro-proliferative effect of the matrices was supported by expression analysis of proliferative markers regulating cell cycle progression. Upregulated expression of genes encoding the adhesive molecules fibronectin, vinculin, CD44 antigen, and the intracellular adhesive molecule-1 was detected in hPDL and hOF cells cultured on each of the four matrices. This may be considered as a prerequisite for good adhesive properties of the four scaffolds ensuring proper cell–matrix and cell–cell interactions. Upregulated expression of genes encoding TGF-β1 and EGF growth factors as well as MMPs in cells grown on each of the four matrices provided support for their pro-proliferative and pro-migratory abilities. The expression of genes encoding the angiogenic factors FGF-2 and VEGF-A was dramatically increased in cells grown on DADM and HADM only, suggesting a good basis for accelerated vascularization of the latter. Altogether, our results support favorable influence of the investigated collagen matrices on the recruitment, attachment, and growth of cell types implicated in oral soft tissue regeneration. Among the four matrices, HADM has consistently exhibited stronger positive effects on the oral cellular behavior. Our data provide solid basis for future investigations on the clinical application of the collagen-based matrices in surgical periodontal therapy.

## 1. Introduction

Various clinical indications such as coverage of gingival recession defects, gain of attached gingiva, and thickening of peri-implant soft tissues alone or in combination with guided bone regeneration require soft tissue surgical reconstructions [1] that depend on the availability of sufficient autologous soft tissue. Harvesting of palatal subepithelial connective tissue grafts or free gingival grafts in sufficient amount and quality cannot always be achieved because of various factors such as shallow palate, thin gingival phenotype, or the need to cover multiple recessions [2,3]. Furthermore, soft tissue graft harvesting requires a second surgical site, thus increasing (1) the patient morbidity, (2) the risk of damaging the palatal artery, (3) the surgical procedure time, (4) the risk of post-operative discomfort and complications, and finally (5) the risk of unpredictable esthetic outcome, e.g., differences in texture and color with adjacent tissues [4,5]. Collagen scaffolds of porcine origin offer a safe alternative to autogenous soft tissue grafts due to their natural origin and excellent biocompatibility, unlimited availability, easy processing, and low cost [6].

A prominent advantage of the naturally derived collagen-based materials is their ability to bind a number of proteins, which makes them a promising carrier of growth factors [7,8,9,10]. In a recent comparative study, we have investigated the ability of four commercially available porcine-derived collagen matrices to adsorb and release growth factors over time [11]. The investigated matrices adsorbed each of the following growth factors, transforming growth factor-β1 (TGF-β1), fibroblast growth factor-2 (FGF-2), platelet-derived growth factor-BB (PDGF-BB), growth and differentiation factor-5 (GDF-5), and bone morphogenetic protein-2 (BMP-2) with a great efficiency. Furthermore, they have shown sustained growth factor release over 13 days with kinetics that will likely favor the long-term tissue regeneration following surgical reconstructive periodontal therapy [11].

The current study extends the in vitro investigations on the four matrices in relation to their ability to modify the behavior of oral cells involved in periodontal regeneration. One of the examined xenografts is a 3D non-crosslinked collagen matrix, which we abbreviated NCM (Geistlich Mucograft^®^; Geistlich, Wolhusen, Switzerland) [12,13]. The NCM comprises two functional layers: a thin compact and a thick porous layer composed of native collagen types I and III. Both surfaces have shown biocompatibility and the ability to support proliferation of gingival fibroblasts, with slightly higher proliferative rates detected on the compact comparted to the spongy layer [14]. A novel sugar-crosslinked collagen matrix, abbreviated CCM (Ossix^®^ Volumax; Datum Dental Ltd., Lod, Israel), has recently emerged as a promising biomaterial for periodontal tissue regeneration. Its production process consists of the extraction of collagen into monomeric fibrils, which are then reconstructed and crosslinked to ribose, a naturally occurring sugar molecule. This novel proprietary crosslinking technology (Glymatrix^®^, Datum Dental Ltd., Lod, Israel) has been shown to increase the barrier capacity of collagen membranes making them impermeable to cellular infiltration over 30 days [15] while allowing the passage of fluids, nutrients, and plasma proteins that support cellular differentiation [16]. Cell–matrix interaction studies have demonstrated that a dry-supplied acellular dermal matrix, which we abbreviated DADM (mucoderm^®^; botiss biomaterials GmbH, Zossen, Germany), supports the metabolic activity and proliferation of human gingival fibroblasts, endothelial cells, osteoblasts, and oral keratinocytes [17]. A recently developed hydrated acellular dermal matrix, abbreviated HADM (NovoMatrix™ Reconstructive Tissue Matrix; BioHorizons, Birmingham, AL, USA), consists of tissue-engineered porcine material that retains components of the natural extracellular matrix (ECM) such as fibronectin, elastin, hyaluronan, and proteoglycans along with an array of fibrillar collagens (types I, II, III, V, XI, XXIV, and XXVII). HADM has shown favorable behavior for treating buccal gingival recession defects in an in vivo animal model compared to NCM [18]. However, thorough analyses of the cellular responses to each of these four matrices are entirely lacking.

The ideal soft tissue substitute is expected to perform a scaffolding function maintaining the biomechanical tissue integrity as well as to improve the natural wound-healing process by triggering its initiation and guiding its progression. On a cellular level, new generation matrices should trigger cellular migration to the wounded area as well as promote cellular adhesion and proliferation [6]. These cellular functions as part of the wound-healing process are rigorously regulated by matrix metalloproteinases (MMPs) and growth factors including TGF-β1, FGF-2, vascular endothelial growth factor-A (VEGF-A), and epidermal growth factor (EGF) [19]. TGF-β1 is recognized as a key regulator of ECM synthesis [20]. FGF-2 plays a role in a number of essential for the wound-healing process events including re-epithelialization, angiogenesis, granulation tissue formation, and tissue remodeling [21]. VEGF-A and EGF are potent stimulators of angiogenesis [22,23]. In addition, EGF induces epithelialization, fibroblast proliferation, and survival [23]. Finally, MMPs degrade ECM components, thus promoting cell migration during tissue remodeling [24].

Oral fibroblasts and periodontal ligament (PDL) cells are two cell types playing a central role in periodontal regeneration [25]. Even though the PDL cell population is predominantly fibroblastic, it includes a small stem cell fraction possessing a high capacity for proliferation, self-renewal, and multilineage differentiation [26,27]. This makes the PDL cell type an interesting model for studying cellular responses toward regenerative biomaterials. The purpose of the present study was to investigate the biological responses of oral fibroblasts and PDL cells to the four commercially available collagen matrices in terms of cellular migration and proliferation induced by the biomaterials. Subsequently, a screen for the expression of proliferative and adhesive gene markers as well as wound healing-related genes was performed.

## 2. Materials and Methods

### 2.1. Cell Culture and Collagen-Based Matrices

Primary human periodontal ligament cells (hPDL) and oral fibroblasts (hOF) were obtained from three donors each using tissue explant technique as described previously [28]. Healthy periodontal ligament derived from the middle third portion of extracted third molars (for obtaining hPDL) or tissue samples harvested from subepithelial palatal mucosa (for obtaining hOF) were retrieved from periodontally and systemically healthy anonymous individuals below 35 years following approval and signed informed consent by the Ethics Committee, Bern, Switzerland (ethical code ID 2018-00661 from 13 August 2018). Tissue samples were minced into 1-mm tissue explant pieces that were transferred to 25 cm^3^ culture flasks and cultured in Dulbecco’s modified Eagle medium (DMEM; Invitrogen, Zug, Switzerland) supplemented with 10% fetal calf serum (FCS; Invitrogen) and 1% antimycotics/antibiotics (AA; ThermoFisher Scientific, Basel, Switzerland). Primary cells that had not undergone more than six passages were starved in 0.3% FCS/DMEM before culturing on collagen matrices.

Geistlich Mucograft^®^ (NCM), Ossix^®^ Volumax (CCM), mucoderm^®^ (DADM), and NovoMatrix™ (HADM), the latter three kindly provided by Regedent AG (Dettelbach, Germany), botiss biomaterials GmbH (Berlin, Germany) and Camlog Biotechnologies GmbH (Basel, Switzerland), respectively, were cut sterile into 10 × 10 mm pieces, rinsed in DMEM for 10 min and placed on the bottom of 24-well ultra-low attachment plates (Corning, NY, USA). Cells seeded in 24-well plates for adherent cell culture (Greiner Bio-One, St. Gallen, Switzerland), without a matrix, were used as control (Ctrl) throughout the study.

### 2.2. Transwell (Boyden Chamber) Cell Migration Assay

Cell migration was assayed using transwell polycarbonate membrane inserts (6.5 mm; Greiner Bio-One) with 8 µm pore size as described [29]. After 24 h of starvation, 5 × 10^4^ cells were plated in the top insert chamber with 200 µL serum-free DMEM. Each of the collagen matrices was placed in the low chamber with 800 µL 10% FCS/DMEM. Cells were allowed to migrate across the filter for 18 h at 37 °C before fixation in Shandon™ Formal-Fixx™ (ThermoFisher Scientific), and staining with 0.1 mg/mL crystal violet (Sigma-Aldrich Chemie GmbH, Buchs, Swtzerland) solution. Images of duplicate inserts were acquired on an Olympus CKX41 microscope equipped with a ProgResCT3 camera. Migration was quantified by using the ImageJ software as described [29]. Data represent means ± SD from three independent experiments performed with three different cell donors for each cell type.

### 2.3. Wound Healing/Cell Migration Assay

Wound healing was assayed using 2-well silicone inserts (ibidi GmbH, Gräfelfing, Germany) placed into a 24-well plate. After 24 h of starvation, 5 × 10^4^ cells/well were plated with 70 µL 10% FCS/DMEM. The cell culture inserts were removed after 24 h leaving a defined cell-free gap of 500 μm. At this time point (0 h), 1.5 mL of 10% FCS/DMEM were added into each well of the 24-well plate and images were taken at 10× magnification. The collagen matrices were then placed over the gap and co-cultured with the cells for 24 h. After careful removal of the matrices, images for the 24 h-time point were captured on a Leica DM IL LED microscope equipped with Leica DFC420 C camera. The cell-free area at 0 (T0) and 24 (T24) h was determined by using ImageJ as described [30]. The percentage of wound closure was calculated using the following formula: [(T0–T24)/T0] × 100. Data represent means ± SD from three independent experiments performed with three different cell donors per cell type, each in triplicates.

### 2.4. Cell Proliferation Assay

Proliferation rates of cultured on the collagen matrices hPDL and hOF cells were determined by trypan blue (Sigma-Aldrich Chemie GmbH) dye exclusion cell counting performed in a Neubauer-improved chamber (Paul Marienfeld, Lauda-Königshofen, Germany). After 24 h of starvation, 5 × 10^3^ cells/well were plated in 3% FCS/DMEM either on collagen matrices (test groups) or on tissue culture plastic (control group) and allowed to proliferate for 11 days. The culture media was replaced every 3 days. Viable cells of each group were counted independently and blindly by two of the co-authors on a Leica DM IL LED microscope on day 1, 3, 5, 7, 9, and 11. Data represent means ± SD from three independent experiments performed with three different cell donors for each cell type.

### 2.5. Quantitative Reverse Transcription-Polymerase Chain Reaction (qRT-PCR) for Gene Expression Analyses

Quantitative RT-PCR was used to investigate the expression of genes encoding (1) proliferative markers (MYBL2, BUB1, PLK1, MKI67, PCNA, CCNE1, CCND1, and CCNB1), (2) adhesive markers (FN1, VCL, CD44, and ICAM1), and (3) wound-healing-related factors (TGFB1, FGF2, VEGFA according to the manufacturer’s protocol). The extracted RNA was additionally purified by using the RNeasy MinElute Cleanup Kit (Qiagen, Basel, Switzerland). RNA, quantified on a NanoDrop 2000c (ThermoFisher Scientific), was reverse transcribed and relative transcripts for the above listed genes, normalized to GAPDH, were measured using FastStart Universal SYBR Green Master ROX (Roche, Basel, Switzerland) and the primer sequences listed in Supplementary Tables S1–S3. Quantitative PCR was carried out in a QuantStudio 3 instrument (Applied Biosystems, Rotkreuz, Switzerland) using a standard thermal cycling profile. The efficiency ∆∆Ct method [31] was used to calculate gene expression levels normalized to GAPDH values and calibrated to values of controls. Samples were run in duplicates. Data represent means ± SD from three independent experiments performed with three different cell donors for each cell type.

### 2.6. Statistical Analysis

All grouped data are means ± SD. Differences between groups were assessed by one-way analysis of variance (ANOVA) with Tukey’s post-hoc test using GraphPad InStat Software (GraphPad, La Jolla, CA, USA), version 3.05. Values of *p* < 0.05 were considered statistically significant.

## 3. Results

### 3.1. Strongly Increased Migration of Primary hPDL and hOF Cells toward Four Differet Collagen Matrices

**Figure 1:**
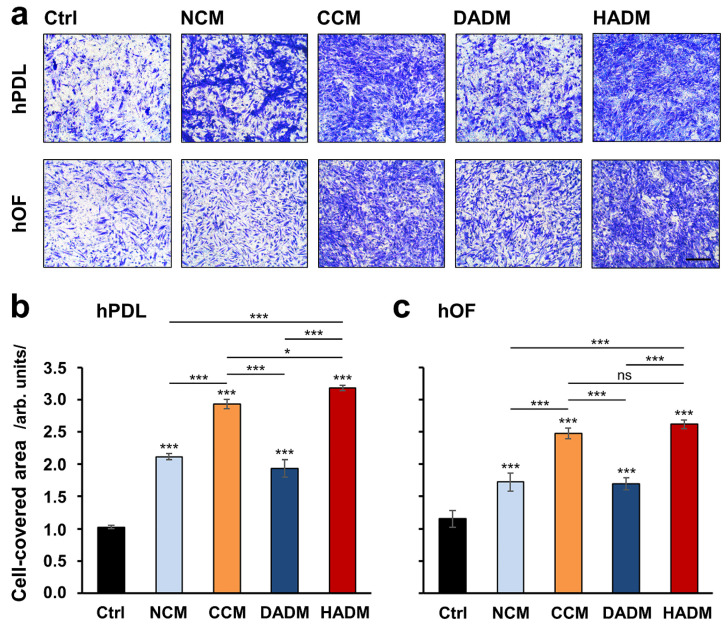
Pro-migratory effects of four porcine-derived collagen matrices on primary human oral cell types. Migration of human periodontal ligament (hPDL) (**a**,**b**) and human oral fibroblasts (hOF) (**a**,**c**) cells toward non-crosslinked collagen matrix (NCM), crosslinked collagen matrix (CCM), dried acellular dermal matrix (DADM), and hydrated acellular dermal matrix (HADM) matrices was evaluated by transwell migration assay using filters with 8 μm pore size. (**a**) Representative images of fixed and stained cells that have migrated to the lower side of the filter in each of the experimental groups. Scale bar, 500 μm. (**b**,**c**) Bar charts presenting quantification of cell migration in the absence (Ctrl) or presence of collagen matrices by measuring the area on the lower side of the filter covered with migrated cells. Data represent means ± SD from three independent experiments performed with three different cell donors, in duplicates. Significant differences to the respective control unless otherwise indicated, *** *p* < 0.001, * *p* < 0.05, ns: not significant.

The migratory capacity of primary hPDL and hOF cells toward four different collagen matrices, indicated for soft tissue augmentation in clinical practice, was examined in vitro by using a transwell migration assay. Each of the collagen matrices significantly induced cell migration by 1.7 to 3.2-fold compared to control cells, where the migration occurred in the absence of a matrix (*p* < 0.001; Figure 1). Among the four investigated matrices, CCM and HADM caused significantly higher increase (*p* < 0.001) in the migration rate of the two cell types. Interestingly, a significant difference in the effects caused by CCM and HADM, in favor of the latter (*p* < 0.05; Figure 1b), was observed in hPDL but not in hOF cells.

### 3.2. Enhanced Wound-Healing Potential of Primary hPDL and hOF Cells Covered with Four Different Collagen Matrices

**Figure 2:**
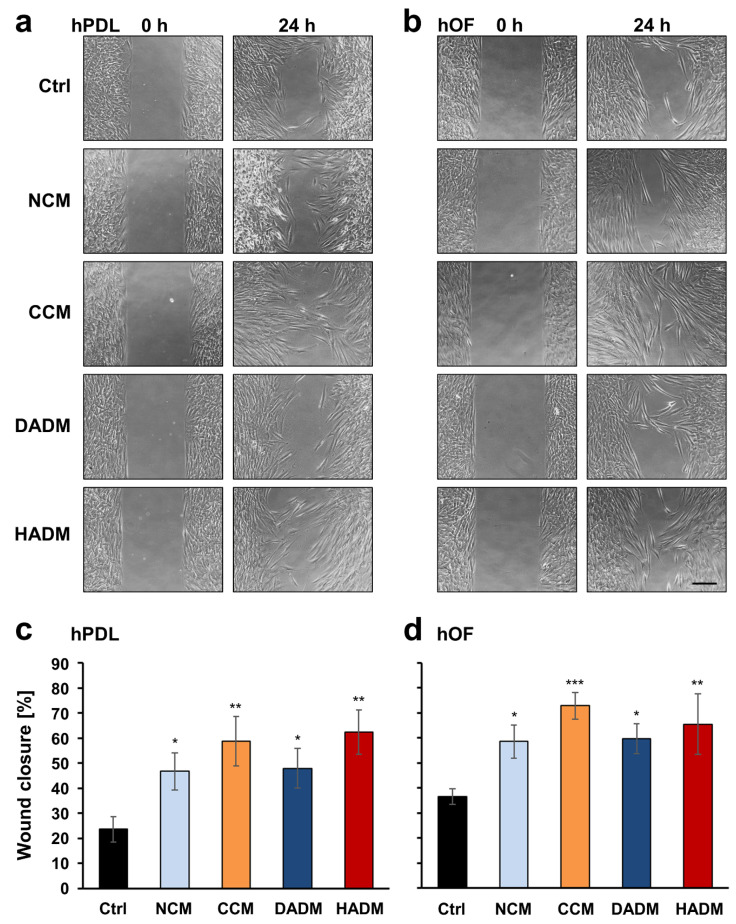
Enhanced wound healing potential of primary human oral cell types covered with four different collagen matrices. (**a**,**b**) Migration of hPDL (**a**) and hOF (**b**) cells toward an wound gap with a 500 µm width generated by seeding cells in a 2-well ibidi culture inserts, in the absence (Ctrl) or in the presence of NCM, CCM, DADM, and HADM matrices placed over the cells. Representative images of the two cell types in each of the experimental groups at 0 and 24 h are shown. Scale bar, 500 μm. (**c**,**d**) Bar charts presenting quantification of the wound healing potential of hPDL (**c**) and hOF (**d**) cells, in the absence or presence of collagen matrices, by measuring the cell-free area at 0 and 24 h. Percentage of wound closure was calculated as described in the Materials and Methods section. Data represent means ± SD from three independent experiments performed with three different cell donors, in triplicates. Significant differences to the control cells, *** *p* < 0.001, ** *p* < 0.01, * *p* < 0.05.

The effects of the different collagen matrices on the wound healing potential of primary hPDL and hOF cells were evaluated by using a modified wound scratch assay. An artificial wound gap with a defined size of 500 µm, which ensures proper comparison between the experimental groups, was generated in a confluent monolayer of either hPDL or hOF cells by using ibidi cell culture inserts. The collagen matrices, placed in contact with the cells at the wound edges for 24 h, were able to increase significantly the capacity of both cell types to migrate into the gap compared to control cells that had no contact with the biomaterial (Figure 2a,b). Compared to a wound closure of 24 and 35% for control hPDL and hOF cells, respectively, NCM and DADM xenografts caused a similar moderate wound closure of 47–48% for hPDL and 59–60% for hOF cells (*p* < 0.05; Figure 2c,d). In contrast, CCM and HADM induced a higher wound coverage of up to 62 and 73% for hPDL and hOF cells, respectively (*p* < 0.01; Figure 2c,d). Although, CCM was able to improve the wound healing potential of hOFs with a great significance compared to the control (*p* < 0.001; Figure 2d), none of the four collagen matrices appeared to cause significantly better effects in terms of wound healing than the others in any of the two oral cell types.

### 3.3. Enhanced Proliferation of Primary hPDL and hOF Cells Cultured on CCM and HADM Collagen Matrices

**Figure 3:**
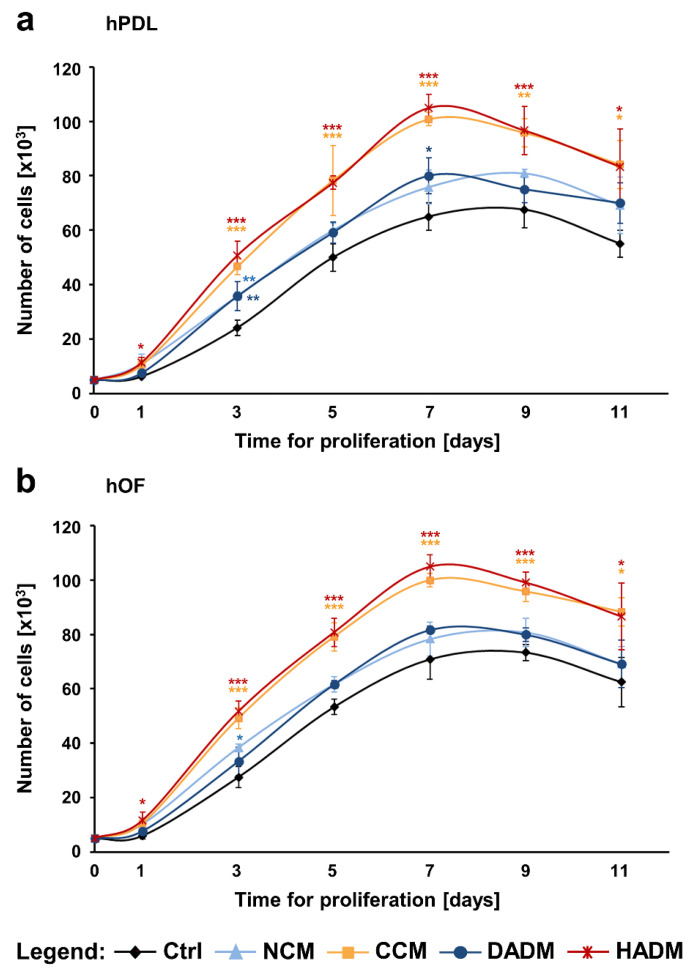
Proliferation rates of primary hPDL (**a**) and hOF (**b**) cultured on tissue culture plastic (Ctrl) or on each of the four collagen matrices (NCM, CCM, DADM, and HADM) were assessed by trypan blue dye exclusion cell counting. The number of viable cells in each experimental group was determined on day 1, 3, 5, 7, 9, and 11. Data represent means ± SD from three independent experiments performed with three different cell donors for each of the two cell types. Significant differences to control cells at each individual time point, *** *p* < 0.001, ** *p* < 0.01, * *p* < 0.05.

The effects of the four collagen matrices on the proliferation potential of primary hPDL and hOF cells were assessed by determining the number of viable cells over 11-day period (Figure 3). The growth curves of cells of both oral cell types, cultured on tissue culture plastic (control) or on each of the four collagen matrices, displayed similar sigmoidal shape with a logarithmic growth between day 2 and 7, suggesting an excellent biocompatibility of the investigated matrices. However, the porous layer of NCM began to degrade after day 5, whereas the other matrices remained compact over the entire 11-day culture period.

Both hPDL and hOF cells grew significantly faster on CCM and HADM compared to control cells, with *p* < 0.001 between day 3 and 9 (Figure 3a,b). In contrast, hPDL and hOF cells seeded on NCM or DADM showed slightly but not significantly higher proliferation rates compared to the proliferation rates of control cells. An exception has been seen on individual time points, e.g., compared to the control, hPDL cells exhibited significantly faster growth on NCM and DADM on day 3 (*p* < 0.01; Figure 3a) and on DADM on day 7 (*p* < 0.05; Figure 3a) whereas significantly faster growth of hOFs was only observed on NCM on day 3 (*p* < 0.05; Figure 3b). Furthermore, compared to NCM and DADM, the pro-proliferative effects of CCM and HADM appeared to be significantly better pronounced in hPDL cells between day 3 and 7 (Figure 3a) as well as in hOFs between day 3 and 9 (Figure 3b). In the sake of a clearer visualization, symbols for significance are not displayed in these latter examples.

### 3.4. Increased Expression of Proliferative Marker Genes in hPDL and hOF Cells Grown on Four Different Collagen Matrices

**Figure 4:**
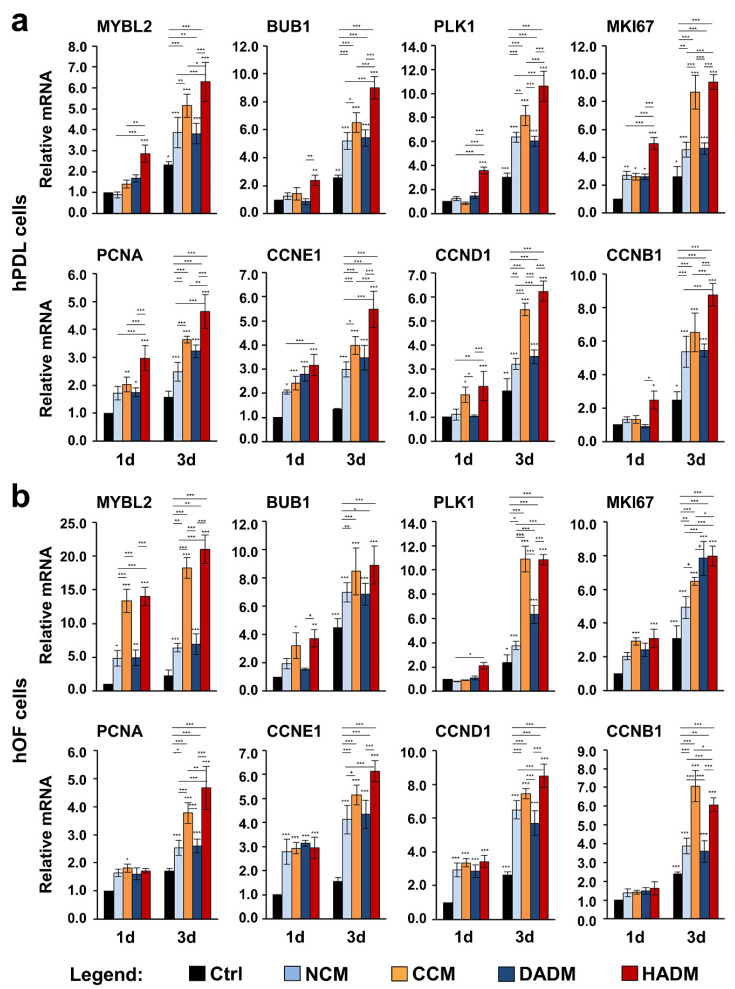
Increased expression of proliferative marker genes in two oral cell types cultured on four different collagen scaffolds. hPDL (**a**) and hOF (**b**) cells were grown on four different collagen matrices (NCM, CCM, DADM, and HADM) for 1 and 3 days before total RNA was isolated and analyzed for the expression of proliferative marker genes (MYBL2, BUB1, PLK1, MKI67, PCNA, CCNE1, CCND1, and CCNB1) by qRT-PCR. Controls (Ctrl) represent cells of each cell type plated on cell culture plastic in the absence of a collagen matrix. Values normalized to GAPDH are expressed relative to the values of control cells at day 1 (1d). Data represent means ± SD from three independent experiments performed with three different cell donors for each of the two cell types. Significant differences to the respective controls at day 1 unless otherwise indicated, *** *p* < 0.001, ** *p* < 0.01, * *p* < 0.05.

To investigate further, how the collagen matrices influence the proliferative capacity of primary hPDL and hOF cells, we tested the expression of genes encoding proliferative markers. These are MYBL2 encoding the Myb-related protein B, BUB1 encoding a mitotic checkpoint serine/threonine-protein kinase, PLK1 encoding the polo-like kinase 1, MKI67 encoding the Ki-67 proliferative marker, PCNA encoding the proliferating cell nuclear antigen, and CCNE1, D1, and B1, encoding cyclin-E1, -D1, and -B1, respectively. All these genes are strictly associated with cell proliferation and most of them appear prominent regulators of the cell cycle progression [32]. A potential dynamics in their expression was analyzed by qRT-PCR on two different time points, 1 and 3 days (Figure 4). On day 1, compared to control hPDL cells seeded in the absence of a collagen matrix, the expression of MYBL2, BUB1, PLK1, CCNB1 was significantly induced in cells seeded on HADM but not on the rest of the matrices (Figure 1a). In hOFs cultured on collagen matrices, PLK1 and CCNB1 showed no difference in their expression compared to control cells on day 1, whereas PCNA was significantly induced in cells seeded on CCM only (Figure 1b). With few exceptions, where a significant induction of proliferative marker gene expression was only seen in cells seeded on CCM in addition to HADM, e.g., CCND1 in hPDL, and BUB1 and MKI67 in hOF cells, prominent upregulation of proliferative markers above their basal expression in control cells on day 1 was triggered by all four collagen matrices. Thus, MKI67 and CCNE1 (Figure 1a) as well as MYBL2, CCNE1, and CCND1 (Figure 1b) were significantly upregulated above two-fold in hPDL and hOF cells, respectively, seeded on all four collagen matrices.

In agreement with the cellular proliferation data as seen in Figure 3, on day 3, we observed a strongly induced expression of all investigated proliferative marker genes in hPDL and hOF cells cultured on each of the collagen matrices compared to the expression levels detected in control cells (Figure 4). As a general trend, the pro-proliferative effects of CCM and HADM appeared to be better pronounced than the pro-proliferative effects of NCM and DADM in both oral cell types (compare orange and red with light and dark blue bars at day 3).

### 3.5. Increased Expression of Adhesive Marker Genes in hPDL and hOF Cells Grown on Four Different Collagen Scaffolds

In the event of disruption of tissue continuity, e.g., during periodontal surgical intervention, new cells must migrate, adhere, and proliferate in order to seal the wound and to initiate an effective regenerative process. To investigate whether the four different collagen matrices may trigger changes in the adhesive properties of the cells grown on them, we investigated the expression of four genes with prominent adhesive functions. These are FN1 encoding fibronectin, VCL encoding vinculin, CD44 encoding the CD44 antigen, and ICAM1 encoding the intercellular adhesion molecule-1. As a matrix glycoprotein, fibronectin is mainly involved in cell-extracellular matrix interactions [33]. As a focal adhesion-specific protein, vinculin is crucial to the structure of focal adhesions and associated with both cell-matrix and cell-cell junctions [34]. As cell surface glycoprotein receptors, the CD44 and the intercellular adhesion molecule-1 also promote a variety of cell–matrix and cell–cell interactions including migration and retention of inflammatory cells to activated endothelium and diseased periodontium [35,36,37,38].

**Figure 5:**
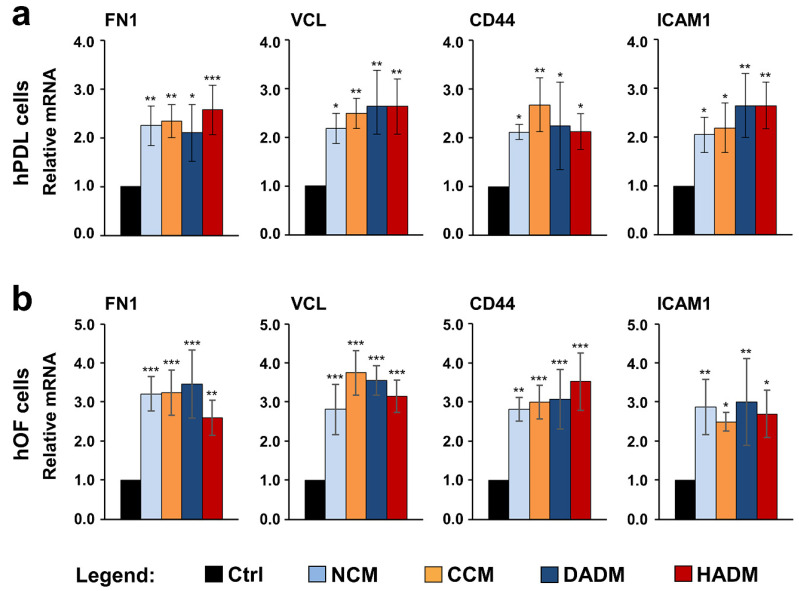
Increased expression of adhesive marker genes in two oral cell types grown on four different collagen matrices. hPDL (**a**) and hOF (**b**) cells were cultured on four different collagen matrices (NCM, CCM, DADM, and HADM) for 9 h followed by an extensive wash for complete removal of nonadherent cells before total RNA was extracted and analyzed for the expression of adhesive marker genes (FN1, VCL, CD44, and ICAM1) by qRT-PCR. Controls (Ctrl) represent cells of each cell type grown on cell culture plastic in the absence of a collagen matrix. Values normalized to GAPDH are expressed relative to the values of control cells. Data represent means ± SD from three independent experiments performed with three different cell donors for each of the two cell types. Significant differences to the respective controls, *** *p* < 0.001, ** *p* < 0.01, * *p* < 0.05.

qRT-PCR analyses revealed a significant upregulation (*p* < 0.05) of all four adhesive genes in both hPDL and hOF cells when they were allowed to adhere on the matrices for 9 h, compared to the expression levels in control cells allowed to adhere on tissue culture plastic, in the absence of a collagen matrix, for the same time (Figure 5). The observed induction in the expression of FN1, VCL, CD44, and ICAM1 was in the range of 2.0–2.7-fold in hPDL (Figure 5a) and 2.5–3.8-fold in hOF cells (Figure 5b). The slightly higher levels of upregulated gene expression in hOFs was due to higher basal expression levels of the four genes in hOF compared to hPDL cells. No significant differences in the potency of the four collagen matrices to induce adhesive marker gene expression in hPDL and hOF cells were detected.

### 3.6. Increased Expression of Wound Healing-Related Genes in hPDL and hOF Cells Grown on Four Different Collagen Matrices

**Figure 6:**
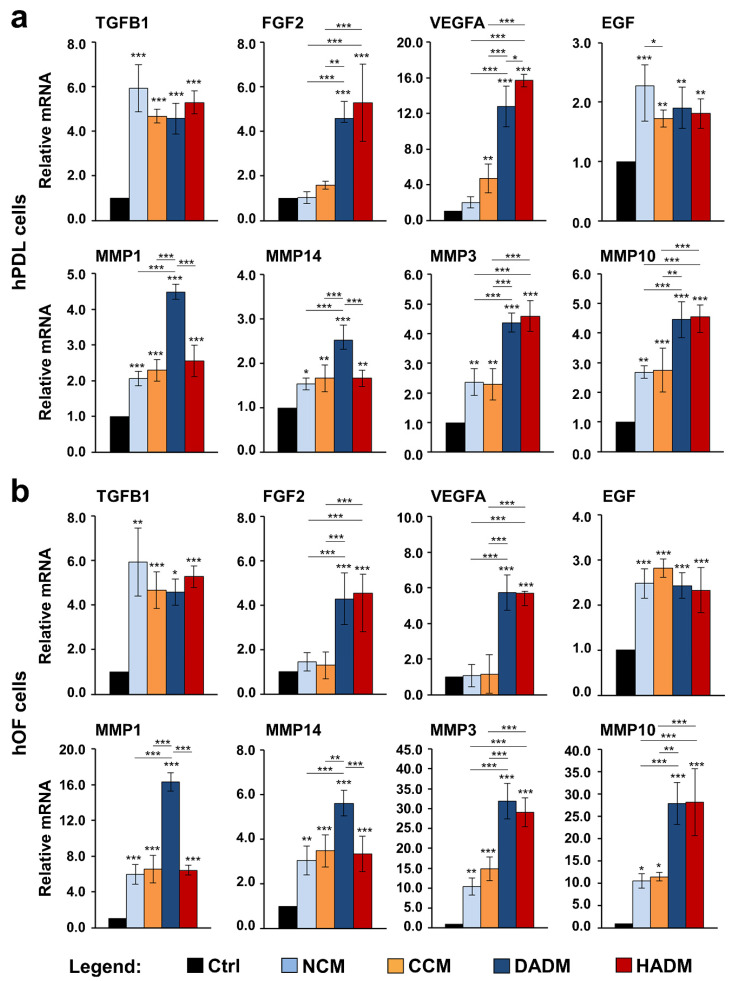
Increased expression of wound healing-related genes in two oral cell types grown on four different collagen matrices. hPDL (**a**) and hOF (**b**) cells were cultured on four different collagen matrices (NCM, CCM, DADM, and HADM) for 24 h before total RNA was isolated and analyzed for the expression of TGFB1, FGF2, VEGFA, EGF, MMP1, MMP14, MMP3, and MMP10 by qRT-PCR. Controls (Ctrl) represent cells of each cell type grown on cell culture plastic in the absence of a collagen matrix. Values normalized to GAPDH are expressed relative to the values of control cells. Data represent means ± SD from three independent experiments performed with three different cell donors for each of the two cell types. Significant differences to the respective controls unless otherwise indicated, *** *p* < 0.001, ** *p* < 0.01, * *p* < 0.05.

Growth factors such as TGF-β1, FGF-2, VEGF-A, and EGF exhibit both individual and synergistic effects on the various stages of the wound healing process, including inflammation and cell recruitment, granulation tissue formation and remodeling [19]. Furthermore, during oral soft tissue healing, collagenases, such as MMP-1 and 14, and stromelysins, such as MMP-3 and 10, continuously remodel the granulation tissue ECM [24,39]. qRT-PCR analyses revealed a significant upregulation of TGFB1, EGF, MMP1, MMP14, MMP3, and MMP10 mRNA levels in both hPDL and hOF cells cultured on each of the four collagen matrices compared to expression levels detected in control cells (Figure 6). With the exception of EGF that appeared slightly but significantly (*p* < 0.05) better expressed in hPDL cultured on NCM compared to hPDL cultured on CCM (Figure 6a), no further differences in the effects caused by the four collagen matrices on the expression of TGFB1 and EGF were detected in the two oral cell types (Figure 6a,b). A very prominent and significantly higher induction of the collagenases MMP1 and MMP14 was characteristic for hPDL and hOF cells cultured on DADM in comparison to the induction observed in the respective cell types cultured on NCM, CCM, or HADM. In contrast, both DADM and HADM caused significantly higher expression of the stromelysins MMP3 and MMP10 in hPDL and hOF cells compared to the moderate induction caused by NCM and CCM. Interestingly, the genes encoding the angiogenesis-related growth factors FGF-2 and VEGF-A showed a significant increase of several fold in hPDL and hOF cells cultured on each of the two acellular dermal matrices DADM and HADM, compared to either control cells cultured in the absence of a biomaterial or cells seeded on NCM or CCM matrices (Figure 6a,b). Concerning the latter, only CCM caused a significant increase in VEGFA mRNA levels above the levels obtained in control hPDL cells (Figure 6a).

## 4. Discussion

Porcine-derived acellular collagen scaffolds have been successfully introduced in the field of periodontal regenerative and implant surgery as a viable alternative to autogenous connective tissue grafts [2,3,6]. The collagenous structure of the regenerative matrices under investigation in the present study contains cellular binding and activation motifs, which are supposed to enhance the recruitment, attachment, and subsequent activation of oral cells with different phenotypes [40]. Therefore, the current study aimed to (1) evaluate the capacity of the four collagen matrices to support migration, adhesion, and growth of primary hPDL and hOF cells, and (2) indirectly elucidate the matrix ability to influence the soft tissue regenerative process by quantifying wound healing-related gene expression that is triggered in cells grown on the matrices. According to the best of our knowledge, this is the first investigation comparing the effects of the four different matrices on the behavior of cells implicated in oral soft tissue regeneration.

To achieve a successful clinical outcome by using resorbable collagen matrices in periodontal plastic surgery, the biomaterial should be populated by cells recruited from the surrounding healthy tissues immediately upon its placement in the defect. This will ensure that the newly formed tissue will successfully replace or integrate with the scaffold before its complete degradation has occurred. Here, we studied the effect of the matrices on the migratory potential of primary hPDL and hOF cells in two different aspects that closely mimic the in vivo clinical situation. On the one hand, the migration of the two oral cell types toward the matrices was investigated by the Boyden chamber migration assay allowing to culture the cells in a close proximity to the matrices and to evaluate their ability to trigger cell migration across a filter from a certain distance. On the other hand, the repopulation of an artificially generated wound gap by the two oral cell types was evaluated by a modified wound scratch assay allowing a direct contact of the cells with the matrices and estimation of the migration potential leading to wound closure. Both scenarios demonstrated highly efficient cell migration triggered by all four matrices with a significantly better-pronounced capability of CCM and HADM to attract cells toward them, compared to NCM and DADM. A possible reason for the observed difference in the efficiency of the matrices to attract oral fibroblasts might be the higher stability of the crosslinked CCM compared to the non-crosslinked counterparts as well as the additional structural components found in the HADM compared to the predominantly collagenous structure of the rest of the matrices. The pro-migratory effect of all four matrices was further supported by the strongly increased expression of genes encoding MMPs in hPDL and hOF cells cultured on the matrices, since the proteolytic activity of the MMPs is directly related to cell migration [41,42].

Once the cells migrate to the augmented site, they must establish proper interactions with the scaffold surface in order to survive, proliferate, and subsequently become activated for executing the next stages of the regenerative process. Efficient cell-matrix adherence serves as a prerequisite for proper cell-matrix interaction since the focal adhesions that the cells form with the underlying matrix surface build not only a tight mechanical attachment but also serve as a biochemical hub triggering inter- and intracellular signaling cascades associated with diverse cellular behaviors [43,44]. Thus, we tested the ability of the four matrices to induce expression of prominent adhesive marker genes such as FN1, VCL, CD44, and ICAM1. Upregulated expression of adhesive markers was detected in hPDL and hOF cells cultured on each of the four matrices, suggesting excellent adhesive properties of the scaffolds. However, microscopy studies are needed to confirm this assumption and to elucidate the attachment of the two cell types to the matrices in a direct way. Interestingly, the upregulated CD44 and ICAM1 genes are involved in regulating diverse cell–cell interactions including migration and recruitment of inflammatory cells to activated endothelium and periodontium [35,36,37,38]. CD44 often binds hyaluronan (HA) and namely the CD44-HA complexes mediate the adhesive interaction between gingival fibroblasts and lymphocytes, which induces signaling cascades regulating cell–cell adhesion, cell migration, and proliferation. Collagens themselves are reported to regulate migration by interacting with various integrins on the cell surface [45]. Thus, future research is needed to investigate whether migration of cells of the immune system, which exhibit inflammatory effects and/or phagocytose oral pathogens, may also be enhanced by these matrices.

Similarly to the favorable effects of CCM and HADM on the cellular motility, the two matrices considerably increased the proliferative rates of hPDL and hOF cells above the rates detected in cells cultured on NCM, DADM, or tissue culture plastic. The ability of an Ossix^®^ collagen membrane to promote adhesion and proliferation of hPDL cells was investigated by Rothamel and colleagues in comparison with collagen membranes such as Bio-Gide^®^, BioMend^®^, and TutoDent^®^ [46]. To the best of our knowledge, no other study has investigated the cellular behavior toward the collagen matrix, which we abbreviated as CCM, from the Ossix^®^ family products. Results from in vivo studies have shown that the degradation of crosslinked collagen membranes is significantly slower compared to non-crosslinked materials [47,48]. However, it is unlikely that the ribose-crosslinking is the only reason for the great pro-proliferative effects exhibited by the CCM, since HADM has triggered comparable cellular proliferative rates under the same conditions. On contrary, cells cultured on NCM and DADM failed to reach the proliferative rates observed on CCM or HADM, even though they showed slightly better proliferation than control cells. The overall positive effect of the matrices on the growth of hPDL and hOF cells as well as the more favorable pro-proliferative impact of CCM and HADM compared to NCM and DADM was further supported by the expression analysis of proliferative marker genes such as MYBL2, BUB1, PLK1, MKI67, PCNA, and CCNE1, CCND1, and CCNB1. These findings agreed with the reports of others who demonstrated no significant differences in the proliferation rate of human gingival fibroblasts cultured on NCM compared to tissue culture plastic [49] or even reduced proliferation on NCM [50]. Numerous clinical studies, however, suggest a positive effect of the NCM and DADM on the soft tissue regeneration [14,51,52,53,54]. Often a direct correlation between in vitro and in vivo studies is not possible due to the fact that cell culture conditions provide for a better control of the variables whereas in vivo conditions comprise structural, cellular, and tissue complexity.

The physicochemical characteristics of the matrices can certainly affect their wound-healing properties [6,55,56]. A number of studies have demonstrated an impact of the matrix composition, porosity and pore size on the cellular attachment, proliferation, and motility. Chen et al. have observed intermediate degrees of spreading and proliferation of vascular smooth muscle cells on an engineered model ECM consisting of fibrillary type I collagen and fibronectin compared to the cellular responses measured on collagen or fibronectin alone [57]. By investigating the migratory and proliferative activity of mesenchymal stem cells on 3D polylactide scaffolds, Rodina et al. have concluded that larger pore size or higher porosity of the scaffold enhances the cellular penetration and growth [58,59]. Chemical, micron- and/or nano-scale topographical analyses of the matrices have not been performed in the present study and deserve detailed investigation. It is probable that namely the porous structure and unique layering of the matrices together with their surface characteristics and motifs involved in the recognition and binding of cells are in the basis of the differential behavior of cells grown on the different matrices. Another factor influencing the cellular response to the matrices is the hydration procedure and time [60]. We have adopted a clinically relevant 10-min washing of the matrices with cell culture medium before cell seeding. Pre-washing of collagen membranes has been recommended in order to enhance their favorable effect on the cellular proliferation [61,62]. Therefore, it is likely that prolonged washing of the matrices may yield different results. However, all four matrices used in the current study showed good cell viability in our experimental setups.

An ideal xenogenic matrix for soft tissue augmentation should not only provide for enhanced cell motility, attachment, and proliferation, but also influence the expression of growth factors and cytokines playing a role at diverse stages of the regenerative process. This led us to investigate the expression of genes encoding TGF-β1, FGF-2, VEGF-A, and EGF growth factors that serve pro-migratory, pro-proliferative, and pro-angiogenic functions at rather early stages of the wound healing process [63] as well as MMPs involved in all regenerative stages and especially during tissue remodeling [24]. The increased TGFB1 and EGF expression observed by us in cells grown on each of the four collagen matrices appears in support of the pro-proliferative effect of all matrices seen in the first days of culturing. Others have also shown an increased TGFB1 gene expression in human gingival fibroblasts grown on polycaprolactone/gelatin nanopolymer scaffolds with incorporated lawsone [64]. Surprisingly, the expression of FGF2 dramatically increased in cells grown on DADM and HADM only. Since the expression of the prominent pro-angiogenic factor VEGFA has nearly mirrored the pattern of expression of FGF2 in cells grown on the different matrices, we can argue that FGF-2 is not indispensable in the early proliferative phase but plays an important role as an angiogenic factor together with VEGF-A. Indeed, FGF-2 injected in the palatal mucosa of rats has promoted vascularization [65] and significantly accelerated the wound healing [66]. Furthermore, crosslinked type I collagen scaffolds loaded with FGF-2 have displayed increased rate of vascularization following submucoperiosteal implantation in the palate of rats [67]. Many growth factors are shown to exhibit synergistic effects on the cellular functions when simultaneously present in the cellular microenvironment. It has been demonstrated that acellular collagen-heparin scaffolds containing both FGF-2 and VEGF can increase angiogenesis and blood vessel maturation following subcutaneous implantation [68].

Our data have shown upregulated MMP gene expression in cells cultured on each of the four matrices with a pattern of expression that closely resembles the one of FGF2 and VEGFA. This may be explained by the fact that the two growth factors may regulate the expression of MMPs, in particular MMP1 and MMP3. FGF-2 is known to enhance MMP1 expression in dermal fibroblasts [21] and MMP3 expression in vascular endothelial cells [69] whereas VEGF upregulates the expression of both MMPs in vascular smooth muscle cells [70]. Whether the increased expression of MMPs in our study is a direct influence of the collagen matrices or an indirect effect, induced by the upregulated expression of genes encoding growth factors, remains to be elucidated.

In conclusion, our data support the notion that the investigated matrices provide a favorable environment able to promote migration, adhesion, and proliferation of both hPDL and hOF cells. Among the four collagen matrices, HADM has consistently exhibited stronger positive effects on the oral cellular behavior suggesting enhanced soft tissue regenerative abilities.

## Supplementary Materials

The following are available online at https://www.mdpi.com/1996-1944/13/17/3819/s1, Table S1: Primer sequences for proliferative marker genes, Table S2: Primer sequences for adhesive marker genes; Table S3: Primer sequences for wound healing-related genes.
