# Supplementary material for: Enhanced Wound Healing Potential of Primary Human Oral Fibroblasts and Periodontal Ligament Cells Cultured on Four Different Porcine-Derived Collagen Matrices
Source: Materials (Basel). 2020 Aug 29;13(17):3819. doi: 10.3390/ma13173819 (PMC7504420; doi:10.3390/ma13173819)
Supplement: Supplementary file 1 [file materials-13-03819-s001.pdf]

materials

Supplementary materials

Enhanced Wound Healing Potential of Primary Human Oral Fibroblasts and Periodontal Ligament Cells Cultured on Four Different Porcine-Derived Collagen Matrices

Zhikai Lin [1,2,3,†], Cristina Nica [1,2,†], Anton Sculean [2,‡] and Maria B. Asparuhova [1,2,*,‡]

[1] Laboratory of Oral Cell Biology, Dental Research Center, School of Dental Medicine, University of Bern, Freiburgstrasse 3, 3010 Bern, Switzerland; zhikai.lin@zmk.unibe.ch (Z.L.); cristina-gabriela.nica@students.unibe.ch (C.N.)
[2] Department of Periodontology, School of Dental Medicine, University of Bern, Freiburgstrasse 7, 3010 Bern, Switzerland; anton.sculean@zmk.unibe.ch
[3] Department of Periodontology, Shanghai Ninth People's Hospital, School of Medicine, Shanghai Jiaotong University, Zhizaoju Road 639, Shanghai 200011, China
* Correspondence: mariya.asparuhova@zmk.unibe.ch; Tel.: +41-31-632-86-22
† Equal contribution.
‡ These authors share senior authorship.

Received: 27 July 2020; Accepted: 26 August 2020; Published: date

Table S1. Primer sequences for proliferative marker genes.

Gene symbol	Primer pair (fwd/rev)
MYBL2	5′-GTCAAATGGACCCATGAGGA-3'
	5′-GTCAGTGCGGTTAGGGAAGT-3'
BUB1	5′-GGAGAACGCTCTGTCAGCA-3'
	5′-TCCAAAAACTCTTCAGCATGAG-3'
PLK1	5′-AACCGAGTTATTCATCGAGACC-3'
	5′-TTGGTTGCCAGTCCAAAATC-3'
MKI67	5′-GAGGTGTGCAGAAAATCCAAA-3'
	5′-CTGTCCCTATGACTTCTGGTTGT-3'
PCNA	5′-ACACTAAGGGCCGAAGATAACG-3'
	5′-ACAGCATCTCCAATATGGCTGA-3'
CCNE1	5′-GGCCAAAATCGACAGGAC-3'
	5′-GGGTCTGCACAGACTGCAT-3'
CCND1	5′-GCCGAGAAGCTGTGCATC-3'
	5′-CCACTTGAGCTTGTTCACCA-3'
CCNB1	5′-CCTCCGGTGTTCTGCTTC-3'
	5′-TTCAGCATTAATTTTCGAGTTCC-3'
GAPDH*	5′-ATCAAGAAGGTGGTGAAGCAG-3'
	5′-TCGTTGTCATACCAGGAAATGAG-3'

*reference gene used for normalization in all qPCR analyses

Table S2. Primer sequences for adhesive marker genes.

Gene symbol	Primer pair (fwd/rev)
FN1	5′-TGCAGGTCCAGATCAAACAG-3'
	5′-TCCACATCAGTGAATGCCAG-3'
VCL	5′-ATGGGTCAAGGGGCATCCT-3'
	5′-GGCCCAAGATTCTTTGTGTAAGT-3'

CD44	5′-CAGCACCATTTCAACCACAC-3'
	5′-CATTTCTGTCTACATCAGTCATCC-3'
ICAM1	5′-CCTTCCTCACCGTGTACTGG-3'
	5′-AGCGTAGGGTAAGGTTCTTGC-3'

Table S3. Primer sequences for wound healing-related genes.

Gene symbol	Primer pair (fwd/rev)
TGFB1	5′-AACCCACAACGAAATCTATGAC-3'
	5′-GGAATTGTTGCTGTATTTCTGG-3'
FGF2	5′-ACATCAAGCTACAACTTCAAGC-3'
	5′-CCGTAACACATTTAGAAGCCAG-3'
VEGFA	5′-ACTGAGGAGTCCAACATCAC-3'
	5′-TCTTTCTTTGGTCTGCATTCAC-3'
EGF	5′-AAGATATACTTTGCCCATACAGCC-3
	5′-AGAGATTTCCCTCTGTCTGTCC-3'
MMP1	5′-TAAAGACAGATTCTACATGCGCAC-3
	5′-AACAGCCCAGTACTTATTCCCT-3'
MMP14	5′-TCAAAGGAGACAAGCATTGGG-3'
	5′-CGGTAGTACTTGTTTCCACGG-3'
MMP3	5′-TTTGCAGTTAGAGAACATGGAG-3'
	5′-ACGAGAAATAAATTGGTCCCTG-3'
MMP10	5′-TGCAGTTAAAGAACATGGAGAC-3'
	5′-CGAGGAATAAATTGGTGCCTG-3'
